# Untargeted Metabolomic Profiling of Liver in a Chronic Intermittent Hypoxia Mouse Model

**DOI:** 10.3389/fphys.2021.701035

**Published:** 2021-07-08

**Authors:** Li-Da Chen, Zhi-Wei Huang, Yu-Zhen Huang, Jie-Feng Huang, Zhong-Ping Zhang, Xue-Jun Lin

**Affiliations:** ^1^Department of Respiratory and Critical Care Medicine, Zhangzhou Affiliated Hospital of Fujian Medical University, Zhangzhou, China; ^2^Department of Otolaryngology, Quanzhou First Hospital Affiliated to Fujian Medical University, Quanzhou, China; ^3^Department of Pathology, Zhangzhou Affiliated Hospital of Fujian Medical University, Zhangzhou, China; ^4^Department of Respiratory and Critical Care Medicine, The First Affiliated Hospital of Fujian Medical University, Fuzhou, China; ^5^Department of Laboratory Medicine, Zhangzhou Affiliated Hospital of Fujian Medical University, Zhangzhou, China

**Keywords:** liver injury, chronic intermittent hypoxia, obstructive sleep apnea, metabolomics, UHPLC/Q-TOF MS

## Abstract

Obstructive sleep apnea (OSA) has been demonstrated to be associated with liver injury. Nevertheless, the mechanisms linking the two disorders remain largely unexplored to date. Based on UHPLC/Q-TOF MS platform, the present study aimed to study the hepatic metabolomic profiling in a chronic intermittent hypoxia (CIH) mouse model to identify altered metabolites and related metabolic pathways. C57BL/6 Mice (*n* = 12 each group) were exposed to intermittent hypoxia or control conditions (room air) for 12 weeks. At the end of the exposure, liver enzymes and histological changes were assessed. Untargeted metabolomics approach by UHPLC/Q-TOF MS and orthogonal partial least squares-discriminant analysis (OPLS-DA) were applied to screen altered metabolites in mice liver. Bioinformatics analyses were applied to identify the related metabolic pathways. CIH treatment caused a remarkable liver injury in mice. A total of 27 differential metabolites in negative ion mode and 44 in positive ion mode were identified between the two groups. These metabolites were correlated to multiple biological and metabolic processes, including various amino acid metabolism, membrane transport, lipid metabolism, carbohydrate metabolism, nucleotide metabolism, ferroptosis, etc. three differential metabolites including glutathione, glutathione disulfide, arachidonic acid (peroxide free) were identified in the ferroptosis pathway. CIH was associated with a significant metabolic profiling change in mice liver. The metabolites in amino acid metabolism, membrane transport, lipid metabolism, carbohydrate metabolism, nucleotide metabolism, and ferroptosis played an important role in CIH-induced liver injury. These findings contribute to a better understanding of the mechanisms linking OSA and liver injury and help identify potential therapeutic targets.

## Introduction

Obstructive sleep apnea (OSA) is a breathing disorder characterized by intermittent nocturnal oxyhemoglobin desaturations and sleep disruption. Obesity, male gender, older age, post-menopausal state, smoking, sedative use, upper airway soft tissue abnormalities, and craniofacial changes are vital risk factors for OSA (Young et al., 2004). It has emerged as a major public health concern due to its high prevalence in mortality and morbidity (Young et al., 1993; Lindberg and Gislason, 2000; Young et al., 2008). The association between OSA and liver diseases has been investigated in previous clinical and animal studies (Savransky et al., 2007a,b; Sundaram et al., 2014). The results suggested that OSA acted as an important risk factor for liver injury, especially non-alcoholic fatty liver disease (NAFLD) (Musso et al., 2012; Mirrakhimov and Polotsky, 2012; Mesarwi et al., 2019).

A meta-analysis including 18 studies with 2183 participants revealed that OSA was correlated to an increased risk of NAFLD, non-alcoholic steatohepatitis, and fibrosis (Musso et al., 2013). Our previous study indicated that OSA severity was associated with elevation of serum aminotransferases and liver steatosis after controlling confounding factors (Chen et al., 2018). A study based on pediatric population suggested that OSA and its related hypoxemia were associated with biochemical and histological measures of NAFLD severity (Sundaram et al., 2014). The mechanisms linking OSA and liver injury have been attributed to different causes, such as oxidative stress, hepatic inflammation, and metabolic disorders (Mirrakhimov and Polotsky, 2012; Paschetta et al., 2015). However, the underlying mechanisms are still being debated.

Metabolomics is a high throughput analytical technique which measures the concentrations of low-molecular-weight metabolites based on either mass spectrometry or nuclear magnetic resonance spectroscopy (Nicholson et al., 1999). Metabolomics provides a unique perspective into biological processes relative to genomics and proteomics as metabolomics identifies what is actually happening in the system (Riekeberg and Powers, 2017). Metabolomics has been widely used to aid in the unraveling of pathophysiological mechanism and identification of potential biomarkers as well as new therapeutic targets for human diseases (Nicholson and Lindon, 2008; Suhre and Gieger, 2012). Ultra-high performance liquid chromatography/quadrupole time-of-flight mass spectrometry (UHPLC/Q-TOF MS) system was widely used because of its better resolution, higher peak capacity and increased sensitivity.

Chronic intermittent hypoxia (CIH) is the primary pathophysiological characteristic of OSA (Dempsey et al., 2010). CIH animal model has been well established and used to study the pathophysiology of OSA. In the present study, we aimed to study the hepatic metabolomic profiling in a CIH mouse model to identify altered metabolites and related metabolic pathways by UHPLC/Q-TOF MS platform. This could provide a new insight into the pathogenesis of OSA-related liver injury.

## Materials and Methods

### Animals and Grouping

Twenty-four wild-type 8-week-old C57BL/6 male mice (body weight, 17–19 g) were purchased from Xiamen University Laboratory Animal Center (Xiamen, China). The mice were housed in cages with a 12-h-day/12-h-night cycle and were freely allowed standard chow and water. All the mice were acclimatized for a week and then randomly assigned to two groups (*n* = 12 each group) using a random number table: CIH and normal control (NC) group. All animal experiments were performed according to the standard ethical guidelines and approved by Xiamen University Animal Care and Use Committee (XMULAC20201002).

### Intermittent Hypoxia (IH) Exposure

Mice in the CIH group were subjected to IH for 12 weeks. The protocol of IH was established according to our previous study (Chen et al., 2020b). The one cycle of IH contained the following elements: oxygen concentration was reduced from 21 to 6% during the first 40 s, stayed at that concentration for 20 s, and then rapidly re-oxygenated to 21% during the subsequent 40 s, stayed at that concentration for another 20s. The mice were exposed to IH for 8 h per day (08:00 a.m. to 04:00 p.m.) during the light phase. NC group mice were kept in room air at the same time. Body weight was monitored weekly for each animal during the whole period of experiment.

### Samples Collection and Tissues Preparation

Before sacrifice, all mice were fasted for 5 h. At the end of the study period, the mice were euthanized by CO_2_ inhalation. Blood draw was performed. The blood samples were collected into centrifuge tubes and centrifuged at 3,000 g for 15 min at 4°C to obtain supernatants for analysis. The liver was quickly removed and washed in phosphate buffer saline. The section of the entire median lobe was placed in 10% neutral buffered formalin. Liver samples from the left lateral liver lobe of each animal were immediately snap-frozen in liquid nitrogen for subsequent metabolomics analyses.

### Biochemical Determinations

Serum aspartate aminotransferase (AST) and alanine aminotransferase (ALT) levels were measured by the standard photometric method using a commercial kit (Jiancheng Bioengineering Institute, Nanjing, China).

### Hematoxylin–Eosin (HE) Staining

The livers were fixed in 10% buffered formalin and then dehydrated in ascending grades alcohol, and embedded in paraffin wax. Then the paraffin embedded samples were serially cut into sections of 5 μm thickness. The liver tissue sections were further hematoxylin dye stained, washed in the running water, then stained with eosin and mounted under coverslips. Histologic examination was performed by a pathologist who was blinded to the group assignment with an Olympus light microscope (Olympus BX50, Tokyo, Japan).

### Metabolite Extractions

One milliliter of cold extraction solvent methanol/acetonitrile/H_2_O (2:2:1, v/v/v) was added to 80 mg sample, and they were adequately vortexed. The lysate was homogenized by MP homogenizer (24 × 2, 6.0 M/S, 60 s, twice) and sonicated at 4°C (30 min/once, twice) then centrifuged at 14,000 g for 20 min at 4°C and the supernatant was dried in a vacuum centrifuge at 4°C. For LC-MS analysis, the samples were re-dissolved in 100 μL acetonitrile/water (1:1, v/v) solvent.

### UHPLC/Q-TOF MS Analysis

Metabolomics analyses were performed on an Agilent 1290 Infinity LC system (Agilent Technologies, Santa-Clara, CA, United States) coupled to an AB SCIEX Triple TOF 6600 System (AB SCIEX, Framingham, MA, United States) with electrospray ionization (ESI) in the negative and positive modes in Applied Protein Technology Co., Ltd. (Shanghai, China). Chromatographic separation was conducted using ACQUITY UPLC BEH Amide 1.7 μm, 2.1 mm × 100 mm column (waters, Ireland). The column temperature was set at 25°C, injection volume was set at 2 μL, and flow rate was set at 0.5 mL/min. The composition of mobile phase was as follows: *A* = 25 mM ammonium hydroxide and equal amount of ammonium acetate in water, *B* = acetonitrile. The flow rate was 0.4 ml/min. The process of linear gradient elution was as follows: 0–0.5 min, 95% B; 0.5–7 min, 95% B to 65% B; 7–8 min, 65% B to 40% B; 8–9 min, stabilized at 40% B; 9–9.1 min, 40% B to 95% B; and 9.1–12 min, stabilized at 95%. Samples were put in automatic sampler at 4°C during the experiment. The quality control samples were utilized to assess the system stability and data quality.

The ESI source conditions were set as follows: Ion Source Gas1, 60 psi; Ion Source Gas2, 60 psi; IonSpray Voltage Floating ± 5,500 V; source temperature, 600°C; TOF MS scan m/z range, 60–1,000 Da; product ion scan m/z range, 25–1,000 Da; TOF MS scan accumulation time, 0.20 s/spectra; product ion scan accumulation time, 0.05 s/spectra. Information dependent acquisition was utilized to acquire the MS/MS spectra under high sensitivity model. The parameters were as follow: declustering potential, ± 60 V; exclude isotopes within 4 Da; Collision Energy, 35 ± 15 eV; candidate ions to monitor per cycle, 10.

### Data Processing and Analysis

The raw MS data in Wiff format were converted into. mzXML format by Proteo Wizard MS Converter tool and then processed by R package XCMS. The process included peak alignment, retention time correction, and peak area extraction. For the metabolomics analysis, normalized data were imported into SIMCA-P (version 14.1, Umea, Sweden) and used for multivariate analysis such as orthogonal partial least-squares discriminant analysis (OPLS-DA) and Pareto-scaled principal component analysis (PCA). The model was validated based on *R*^2^ and *Q*^2^-values and the results of permutation experiments. Q^2^ represents the prediction performance of the model, and R^2^ represents the percentage of the variable that the model can interpret in a certain direction. The more Q^2^ is closer to 1, the better the model. The combination of Student’s *t-*test (*p-*value) on the data and statistically significant thresholds of variable importance in the projection (VIP) values obtained from OPLS-DA model was applied to identify the differential metabolites. The *p*-values less than 0.05 and VIP values larger than 1.0 were regarded as significant. Kyoto Encyclopedia of Genes and Genomes (KEGG) was used to analyze the relevant pathways enriched by metabolites.

### Statistical Analysis

Data were presented as means ± standard deviation (SD). Student’s *t*-test was adopted to assess significant differences between two groups. GraphPad Prism version 8.0 was used for the statistical analysis (GraphPad Software Inc., San Diego, CA, United States). The *p*-value was considered significant if it is less than 0.05.

## Results

### Effect of CIH on Body Weight, Liver Enzymes, and Liver Histology

**FIGURE 1:**
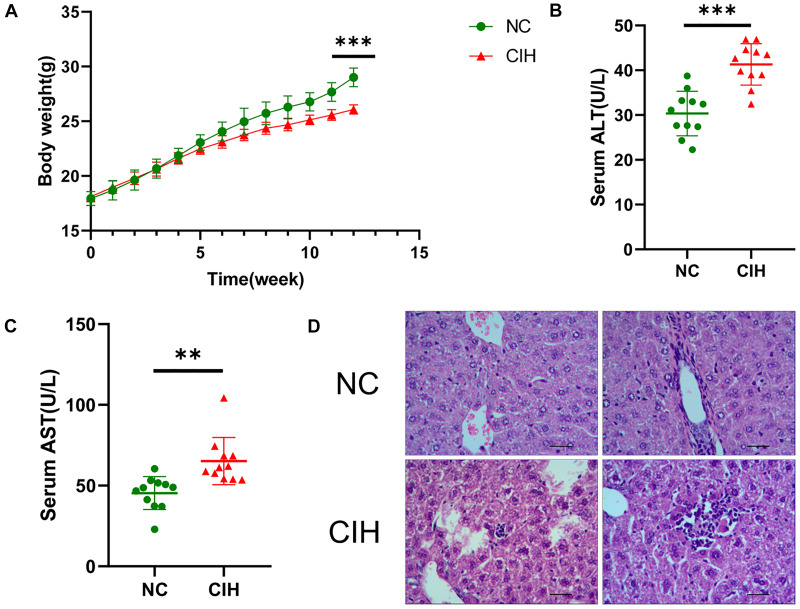
Effect of CIH on body weight, liver enzymes, and liver histology. **(A)** The body weight of mice in CIH group is significantly lower than that in NC group after 12 weeks of exposure. **(B)** Both serum ALT and **(C)** AST levels of mice in CIH group are significantly higher than those in NC group (*n* = 11 each group). **(D)** Hepatic morphology was found to be normal in the NC group with no obvious lobular necrosis or portal inflammation (original magnification, isd t; scale bar, 50 ic). The liver tissue of CIH group revealed lobular necrosis and portal inflammation (original magnification, group scale bar, 50 μs). ^∗∗^*p* < 0.01; ^∗∗∗^*p* < 0.001.

As shown in Figure 1A, no significant difference was noted in the baseline body weight between two groups (17.94 ± 0.64 vs. 18.10 ± 0.48 g, *p*0.05), the body weight of mice was statistically lower in CIH group than that in NC group after 12 weeks of exposure (29.01 ± 0.86 vs. 26.08 ± 0.42 g, *p* < 0.001). In addition, CIH resulted in significant elevation in both serum ALT (30.34 ± 4.97 vs. 41.30 ± 4.62 U/L, *p* < 0.001) and AST (45.35 ± 10.18 vs. 65.19 ± 14.62 U/L, *p* < 0.01) (Figures 1B,C). The liver histology was further evaluated. Hepatic morphology was found to be normal in the NC group with no obvious lobular necrosis or portal inflammation (Figure 1D). The liver tissue of CIH group revealed lobular necrosis and portal inflammation (Figure 1D).

### Quality Control of UHPLC-Q-TOF/MS Analysis

Supplementary Figures 1A,B showed the total ion chromatograms of the quality control sample. It revealed that the variation result from instrumental error was small during the analysis as the retention time and response strength of each chromatographic peak almost overlapped. The Pearson correlation analysis results demonstrated that the repeatability of experiment was good as the correlation coefficients between quality control samples are all above 0.9 (Supplementary Figures 1C,D). There is general consensus that an RSD of the quality control samples < 0.3 is indicative of good reproducibility. More than 80 percent of peaks in this experiment had an RSD < 0.3. This proved that the instrument analysis system was stable (Supplementary Figures 1E,F). In addition, Hotelling’s T2 analysis showed that all the samples in this study were all within the 99% confidence interval, confirming a good repeatability (Supplementary Figures 1G,H). Multivariate control chart showed that the score values of all samples fell within ± 3SD, reflecting that the instrument volatility was mild (Supplementary Figures 1I,J). The above results suggested that the instrument analysis system was stable and the data were reliable.

### Multivariate Analysis of UHPLC-Q-TOF/MS Data

**FIGURE 2:**
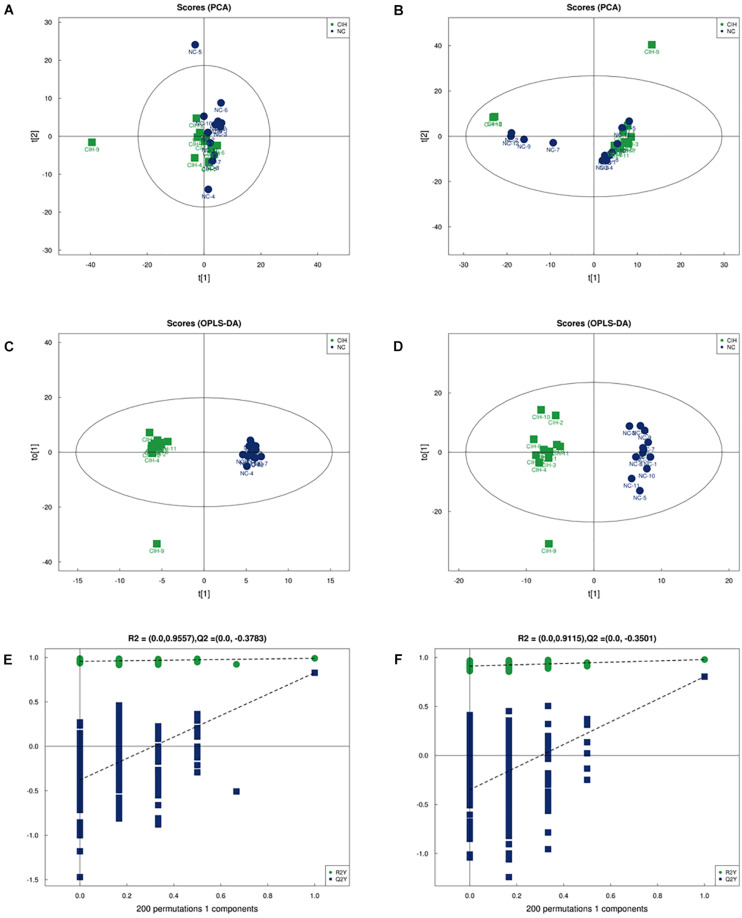
Multivariate analysis of UHPLC-Q-TOF/MS data **(A)** Score plot of PCA in positive ion mode (*R^2^X* = 0.546) and **(B)** negative ion modes (*R^2^X* = 0.579). **(C)** Score plot of OPLS-DA in positive ion mode (*R^2^X* = 0.506, *R^2^Y* = 0.99, and *Q*^2^ = 0.828) and **(D)** negative ion mode (*R^2^X* = 0.614, *R^2^Y* = 0.978, and *Q*^2^ = 0.804). **(E)** Permutation test results of the OPLS-DA model in the positive ion mode and **(F)** negative ion mode. The *Q*^2^-value represents the predictability of the model. The R^2^Y value represents the goodness of fit of the model. All R^2^Y and Q^2^ values to the left are lower than the original points to the right, suggesting that the OPLS-DA model is valid.

The comparison between CIH group and NC group was performed based on multivariate statistical analysis including PCA and OPLS-DA techniques to examine clusters for each group. The PCA analysis showed that the metabolomic profiles of CIH mice were distinctly separated from NC mice for both positive (*R*^2^*X* = 0.546, Figure 2A) and negative ion modes (*R*^2^*X* = 0.579; Figure 2B). This suggested that CIH induced significant changes in the biochemical metabolites in mice liver. In addition, the differences between the CIH and NC mice were identified by the supervised OPLS-DA model. Figures 2C,D showed the OPLS-DA scores plot. A clear distinction was noted between CIH and NC mice for both positive (*R*^2^*X* = 0.506, *R*^2^*Y* = 0.99, *Q*^2^ = 0.828) and negative ion modes (*R*^2^*X* = 0.614, *R*^2^*Y* = 0.978, *Q*^2^ = 0.804). Figures 2E,F presented the permutation test plot. All permutated values were lower than the original values at the right and the line plot intercepted *Y*-axis below zero, this suggested that the original OPLS-DA model was valid and not over-fitted.

### Univariate Analysis of UHPLC-Q-TOF/MS Data

**FIGURE 3:**
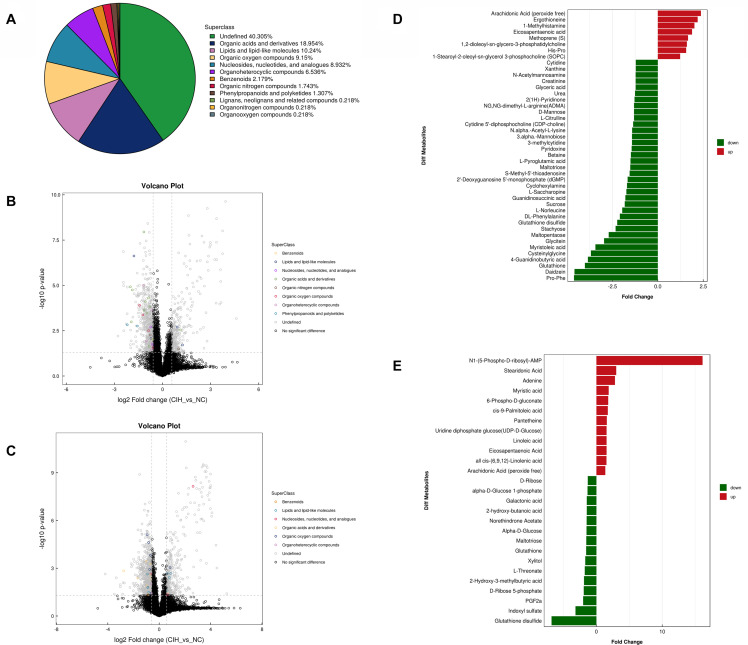
Univariate analysis of UHPLC-Q-TOF/MS data **(A)** Superclass pie chart of differential metabolites between NC and CIH group. **(B)** Volcano plot in positive ion mode and **(C)** negative ion mode. Each point in the volcano plot represents a metabolite. Differentially abundant metabolites of different categories are individually color coded. **(D)** Histogram of significantly differentially detected metabolites in positive ion mode and **(E)** negative ion mode. The *x*-axis shows the fold change; the *y*-axis corresponds to the differential metabolites. Green and red indicate decreased and increased differential metabolites, respectively.

**FIGURE 4:**
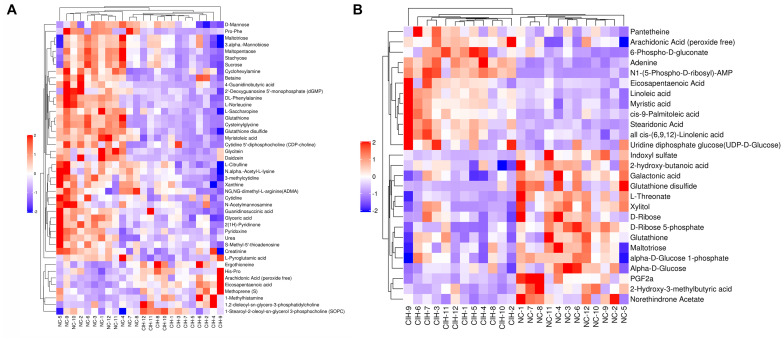
The metabolite heatmaps between NC and control group **(A)** negative ion mode; **(B)** positive ion mode.

The variable VIP generated by OPLS-DA analysis was used to select those metabolites with obvious discrimination potential. The higher VIP score of metabolites means a greater discrimination potential. Based on the criteria of *p* < 0.05 and VIP > 1, a total of 44 differential metabolites in positive ion mode (Table 1) and 27 in negative ion mode (Table 2) were identified in the mice livers between CIH group and NC group. Volcano plots were drawn according to the *p*-value (< 0.05) and fold change (> 1.5 or < 0.67) to visualize dysregulated metabolites between the two groups (positive ion mode, Figure 3A; negative ion mode, Figure 3B). As shown in the histogram in Figure 3C, the top 5 upregulated metabolites in positive ion mode were arachidonic acid (peroxide free), ergothioneine, 1-methylhistamine, eicosapentaenoic acid, and methoprene(S); the top five downregulated metabolites in positive ion mode were pro-phe, daidzein, glutathione, 4-guanidinobutyric acid, and cysteinylglycine. As shown in the histogram in Figure 3D, the top 5 upregulated metabolites in negative ion mode were N1-(5-phospho-D-ribosyl)-AMP, stearidonic acid, adenine, myristic acid, and 6-phospho-D-gluconate; the top 5 downregulated metabolites in negative ion mode were glutathione disulfide, indoxyl sulfate, PGF2α, D-ribose 5-phosphate, and 2-hydroxy-3-methylbutyric acid (Figure 3E). The metabolite heatmaps were also performed to analyze the metabolic pattern in different groups. The results were showed in the Figure 4.

**TABLE 1:** The differential metabolites in positive ion mode.

**ID**	**Adduct**	**Name**	**VIP**	**Fold change**	***p*-value**	**m/z**	**rt(s)**
M269T33	(M + H-2H_2_O) +	Arachidonic acid (peroxide free)	5.083	2.374	0.019	269.226	33.143
M230T170	(M + H) +	Ergothioneine	1.804	2.190	0.003	230.095	169.678
M126T339	(M + H) +	1-Methylhistamine	1.105	2.011	0.032	126.102	339.043
M285T37_2	(M + H-H2O) +	Eicosapentaenoic acid	4.423	1.881	0.002	285.220	37.238
M311T202_2	(M + H) +	Methoprene (S)	1.309	1.664	0.033	311.257	201.812
M831T89	(M-H + 2Na) +	1,2-dioleoyl-sn-glycero-3-phosphatidylcholine	6.602	1.598	0.045	830.566	88.508
M253T365	(M + H) +	His-Pro	1.254	1.557	0.026	253.128	364.791
M855T138	(M-2H + 3Na) +	1-Stearoyl-2-oleoyl-sn-glycerol 3-phosphocholine (SOPC)	2.062	1.227	0.030	854.566	138.009
M244T242	(M + H) +	Cytidine	1.180	0.824	0.026	244.092	242.405
M153T224_2	(M + H) +	Xanthine	2.644	0.821	0.004	153.040	223.609
M204T260	(M + H-H2O) +	N-Acetylmannosamine	1.505	0.820	0.028	204.086	260.007
M114T173	(M + H) +	Creatinine	1.566	0.817	0.017	114.066	172.571
M106T65	M +	Glyceric acid	1.028	0.817	0.004	106.028	64.954
M61T105	(M + H) +	Urea	1.691	0.799	0.037	61.039	104.913
M96T64	(M + H) +	2(1H)-Pyridinone	1.427	0.779	0.004	96.044	64.371
M203T500	(M + H) +	NG,NG-dimethyl-L-arginine(ADMA)	8.783	0.763	0.011	203.150	499.625
M198T301	(M + NH4) +	D-Mannose	1.271	0.763	0.036	198.096	300.642
M176T390	(M + H) +	L-Citrulline	1.047	0.762	0.001	176.102	389.846
M489T440	(M + H) +	Cytidine 5′-diphosphocholine (CDP-choline)	1.322	0.735	0.023	489.112	439.942
M189T387_2	(M + H) +	N.alpha.-Acetyl-L-lysine	1.204	0.708	0.002	189.122	387.197
M343T453	(M + H) +	3.alpha.-Mannobiose	1.291	0.705	0.031	343.122	452.691
M258T240	(M + H) +	3-methylcytidine	1.185	0.705	0.000	258.108	239.746
M170T109	(M + H) +	Pyridoxine	1.286	0.693	0.034	170.080	109.033
M118T274	(M + H) +	Betaine	12.240	0.676	0.040	118.087	274.486
M147T372_2	(M + NH4) +	L-Pyroglutamic acid	3.712	0.666	0.010	147.076	372.230
M522T453	(M + NH4) +	Maltotriose	7.116	0.660	0.027	522.201	453.306
M298T102	(M + H) +	S-Methyl-5′-thioadenosine	2.713	0.646	0.018	298.096	102.332
M389T454	(M + CH3CN + H) +	2′-Deoxyguanosine 5′-monophosphate (dGMP)	1.497	0.602	0.002	389.102	454.037
M160T384_2	(M + CH3COO + 2H) +	Cyclohexylamine	10.006	0.589	0.005	160.133	383.530
M277T448	(M + H) +	L-Saccharopine	5.345	0.578	0.000	277.139	447.933
M176T402	(M + H) +	Guanidinosuccinic acid	2.104	0.561	0.023	176.066	402.419
M325T496	(M + H-H2O) +	Sucrose	1.090	0.549	0.003	325.112	496.495
M173T245	(M + CH3CN + H) +	L-Norleucine	1.378	0.510	0.000	173.128	245.287
M207T234	(M + CH3CN + H) +	DL-Phenylalanine	1.137	0.479	0.000	207.112	233.869
M613T495	(M + H) +	Glutathione disulfide	16.603	0.448	0.000	613.158	495.407
M667T497	(M + H) +	Stachyose	3.304	0.430	0.000	667.226	496.564
M829T516	(M + H) +	Maltopentaose	1.468	0.370	0.000	829.279	515.888
M285T40	(M + H) +	Glycitein	1.215	0.338	0.002	285.075	40.389
M209T35_2	(M + H-H2O) +	Myristoleic acid	3.614	0.291	0.000	209.189	34.796
M179T400	(M + H) +	Cysteinylglycine	2.825	0.272	0.000	179.048	399.855
M146T358_3	(M + H) +	4-Guanidinobutyric acid	4.682	0.260	0.001	146.092	358.152
M308T400	(M + H) +	Glutathione	10.507	0.249	0.000	308.090	399.883
M255T40	(M + H) +	Daidzein	1.626	0.218	0.001	255.064	39.850
M263T478	(M + H) +	Pro-Phe	1.170	0.217	0.000	263.134	478.113

**TABLE 2:** The differential metabolites in negative ion mode.

**ID**	**Adduct**	**Name**	**VIP**	**Fold change**	***p*-value**	**m/z**	**rt(s)**
M558T439	(M-H)-	N1-(5-Phospho-D-ribosyl)-AMP	18.163	16.122	0.000	558.063	439.311
M275T40	(M-H)-	Stearidonic acid	2.444	3.016	0.000	275.200	40.431
M134T325	(M-H)-	Adenine	1.006	2.818	0.000	134.046	324.707
M227T46	(M-H)-	Myristic acid	5.610	1.864	0.002	227.201	45.512
M257T446	(M-H2O-H)-	6-Phospho-D-gluconate	1.089	1.795	0.001	257.005	445.675
M253T44	(M-H)-	*cis*-9-Palmitoleic acid	12.864	1.733	0.004	253.217	43.587
M277T66	(M-H)-	Pantetheine	1.083	1.580	0.033	277.121	66.107
M565T443	(M-H)-	Uridine diphosphate glucose(UDP-D-Glucose)	1.305	1.553	0.045	565.045	442.545
M279T40	(M-H)-	Linoleic acid	27.592	1.545	0.002	279.233	40.400
M301T40	(M-H)-	Eicosapentaenoic acid	4.659	1.540	0.026	301.216	39.915
M277T40	(M-H)-	all *cis*-(6,9,12)-Linolenic acid	7.534	1.522	0.002	277.216	40.438
M303T186	(M-H)-	Arachidonic acid (peroxide free)	2.075	1.352	0.004	303.231	185.744
M209T283	(M + CH3COO)-	D-Ribose	1.501	0.762	0.036	209.066	283.403
M259T454	(M-H)-	alpha-D-Glucose 1-phosphate	4.254	0.745	0.029	259.022	454.500
M195T371	(M-H)-	Galactonic acid	5.052	0.684	0.001	195.051	371.126
M103T190	(M-H)-	2-hydroxy-butanoic acid	1.267	0.683	0.008	103.039	189.710
M339T27	(M-H)-	Norethindrone acetate	1.902	0.678	0.007	339.199	27.430
M179T261	(M-H)-	Alpha-D-Glucose	3.052	0.670	0.016	179.055	261.404
M563T452	(M + CH3COO)-	Maltotriose	3.098	0.648	0.036	563.180	452.109
M306T397	(M-H)-	Glutathione	12.142	0.645	0.036	306.076	396.567
M211T329	(M + CH3COO)-	Xylitol	2.160	0.594	0.001	211.082	328.536
M135T327_2	(M-H)-	L-Threonate	2.564	0.567	0.000	135.029	327.240
M117T154	(M-H)-	2-Hydroxy-3-methylbutyric acid	1.282	0.530	0.002	117.055	153.871
M289T457_2	(M + CH3COO)-	D-Ribose 5-phosphate	3.136	0.521	0.000	289.032	456.644
M353T177	(M-H)-	PGF2a	2.229	0.499	0.001	353.231	177.495
M212T29	(M-H)-	Indoxyl sulfate	4.138	0.315	0.004	212.002	28.760
M611T580	(M-H)-	Glutathione disulfide	3.991	0.147	0.001	611.141	579.775

### Metabolic Pathway Analysis

**FIGURE 5:**
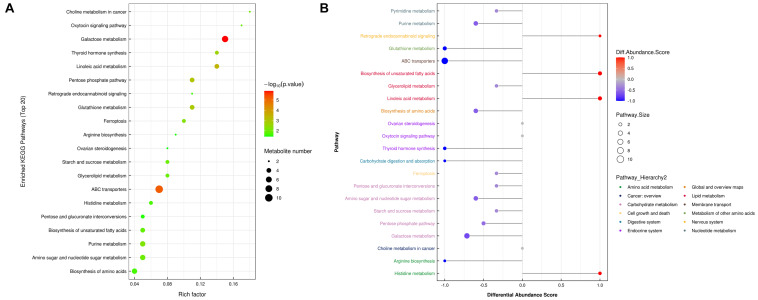
Metabolic pathway analysis. **(A)** Analyses of KEGG pathway enrichment. The *x*-axis shows the enrichment factor; the *y*-axis corresponds to the KEGG pathway. The size of the dot represents the number of metabolites mapped to the reference pathways. The color of the dot represents the *p*-value. **(B)** A pathway-based analysis of metabolic changes upon comparing NC with CIH group. The differential abundance score captures the average, gross changes for all metabolites in a pathway. A score of 1 indicates all measured metabolites in the pathway increase, and -1 indicates all measured metabolites in a pathway decrease. The differential color of pathway is mapped into corresponding category of pathway hierarchy.

**FIGURE 6:**
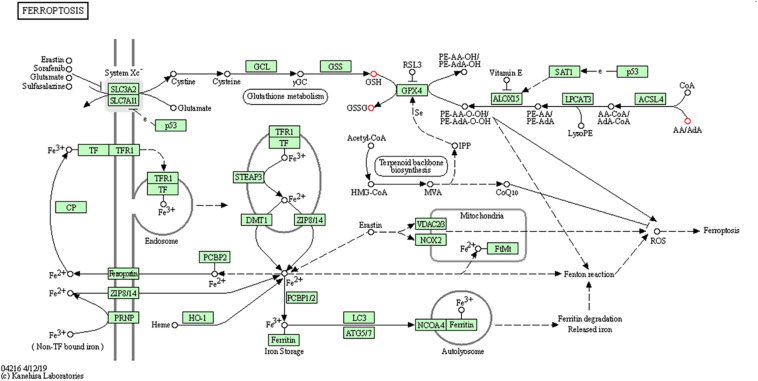
The KEGG map of ferroptosis pathway The KEGG map shows metabolic, signaling, and molecular interaction networks. The red dot represents the differential metabolite.

To further determine which metabolism pathways were affected, we performed pathway analysis based on KEGG using Fisher’s exact test. These identified metabolic pathways were involved in cell growth and death, lipid metabolism, amino acid metabolism, carbohydrate metabolism, membrane transport, nucleotide metabolism, etc. (Figures 5A, B). It was worth noting that the ferrroptosis pathway played a role in the mechanisms of CIH-induced liver injury. Three differential metabolites including glutathione, glutathione disulfide, arachidonic acid (peroxide free) were identified in the ferroptosis pathway. The KEGG map in Figure 6 presented metabolic, signaling, and molecular interaction networks of ferroptosis pathway.

## Discussion

In this study, we established a CIH mouse model to explore the pathophysiological mechanisms underlying CIH-induced liver injury. A UHPLC-Q-TOF MS/MS-based metabolomics approach was utilized to explore the metabolic signatures of liver in a CIH mouse model. Some differential metabolites with 44 in positive ion mode and 27 in negative ion mode were identified between the two groups. These identified metabolic pathways were involved in various biological and metabolic processes, including various amino acid metabolism, membrane transport, lipid metabolism, carbohydrate metabolism, nucleotide metabolism, etc. The ferrroptosis pathway was also identified to play a role in the mechanisms of CIH-induced liver injury.

The effect of CIH on the liver has been assessed in several previous animal studies. As early as 2007, Savransky et al. (2007b) demonstrated that CIH resulted in liver injury and increased levels of oxidative stress and inflammatory markers in the liver in a CIH animal model. Subsequently, in 2009, the same researchers found that CIH and chronic acetaminophen treatment greatly enhanced hepatic toxicity of chronic acetaminophen administration, resulting in liver injury, liver necrosis, and liver inflammation (Savransky et al., 2009). Another study revealed that CIH combined with high fat condition caused more severe injury to the hepatic histology, liver function, and ultrastructure than those of single high fat condition. With the increase of IH exposure time, the liver injury became more severe (Feng et al., 2011). The metabolic characteristics during CIH-induced liver injury were unknown. Unraveling them is helpful to better understand the pathophysiological mechanisms of OSA-related liver injury.

Metabolomics, as a high throughput analytical method, is effective in identifying alteration of metabolites after occurrence of human diseases. It can provide insight into the metabolic processes and pathogenesis of human diseases from a systematic perspective. A few studies used this approach to study the association between OSA and the change of metabolites (Ferrarini et al., 2013; Xu et al., 2016; Alterki et al., 2020; Humer et al., 2020; Reutrakul et al., 2021). Ferrarini et al. (2013) collected plasma samples of 33 OSA patients and analyzed them with LC-QTOF-MS. They reported that the OSA severity was related to the different patterns of molecules. Another study including obese pregnant women diagnosed with gestational diabetes mellitus reported that the metabolomics profiles in OSA group were similar to non-OSA group. Nevertheless, 12 features, annotated to lysophospholipids, estradiols, and fatty acids, were significantly associated with OSA severity (Ferrarini et al., 2013). As far as we know, there is no study utilized this methodology to explore the pathophysiology of OSA in a CIH animal model.

In this study, the differential metabolites were involved in various amino acid metabolism, membrane transport, lipid metabolism, carbohydrate metabolism, nucleotide metabolism. The results were supported by a previous study (Li et al., 1985). Li et al. (1985) reported that the liver triglyceride and phospholipid content of obese mice in CIH group were statistically higher than those in NC group. Further gene expression analysis demonstrated that CIH could upregulate the pathways of lipid biosynthesis in the liver. Our previous study used Illumina HiSeq 4000 platform to explore the dysregulated genes between CIH and NC rats. The results also suggested that CIH resulted in the change of gene expression profiles, and the dysregulated genes were enriched in the metabolic pathways, carbon metabolism, fatty acid metabolism, etc. (Chen et al., 2020a).

We found that ferroptosis pathway was identified to have role in the mechanisms of CIH-related liver injury in this study. Ferroptosis is a new type of lipid- and iron-dependent regulated cell death caused by glutathione depletion and production of lipid peroxides by lipoxygenase enzymes (Dixon et al., 2012). The role of ferroptosis in CIH-induced liver injury was further supported by our previous study. It showed that CIH treatment resulted in significant ferroptosis in rat liver, evidenced by increase of lipid peroxidation, reduced expression of GPX4, increased expression of ACSL4 (Chen et al., 2020b). Furthermore, accumulating evidence demonstrated that ferroptosis implicated in the pathogenesis of multiple organ injury induced by ischemia/reperfusion injury (Fang et al., 2019; Li et al., 2019), which closely mimics physiology of intermittent hypoxia. Glutathione depletion is one of the important characteristics of the ferroptosis (Stockwell et al., 2017). In this study, three differential metabolites including glutathione, glutathione disulfide, arachidonic acid (peroxide free) were identified in the ferroptosis pathway. Savransky et al. (2009) also demonstrated that CIH combined with acetaminophen treatment group exhibited lower hepatic glutathione than acetaminophen treatment group. Future studies are needed to evaluate whether targeting ferroptosis can alleviate CIH-induced liver injury.

Our study has some strengths warrant further comment. As far as we know, this is the first study utilized metabolomics approach to study the pathophysiology of CIH-induce liver injury. In addition, the quality control analysis indicated that all data were credible. So the differential metabolites can reflect the differences between the two groups objectively.

There are some limitations should be acknowledged. Firstly, the CIH model mimics only one of the major characteristics of OSA. However, other factors associated with OSA such as sleep fragmentation, sympathetic hyperactivity, and hypercapnia might participate in OSA-related liver injury. Secondly, some other metabolites were detected in this study, but remained unidentified at present. Thirdly, metabolic changes were investigated only in liver. The serum sample was not investigated simultaneously. Fourthly, some researchers regard the metabolites as the real end points of gene expression. However, the liver is a highly heterogeneous organ, and the signal pathway involved in the differential metabolites may not represent all metabolic flux variations. Fifthly, there was a significant difference in body weight between NC and CIH group after treatment, which might have impact on liver metabolites. Finally, the dysregulated metabolites identified in this study may contribute to CIH-induced liver injury, but the exact molecular mechanisms still needs further study to make them clear. Other markers of ferroptosis including ROS and the changes of mitochondria determined by transmission electron microscopy were not evaluated in this study.

## Conclusion

In conclusion, CIH treatment caused a remarkable liver injury in mice. CIH was associated with a significant metabolic profiling change in mice liver. The dysregulated metabolites were involved in various amino acid metabolism, membrane transport, carbohydrate metabolism, lipid metabolism, nucleotide metabolism, ferroptosis. These findings provided an overall mechanistic view of the metabolic changes during CIH-induced liver injury, which could expand our understanding of the mechanisms of OSA-related liver injury and help identify potential therapeutic targets.

## Data Availability Statement

The original contributions presented in the study are included in the article/Supplementary Material, further inquiries can be directed to the corresponding author/s.

## Ethics Statement

The animal study was reviewed and approved by the Xiamen University Animal Care and Use Committee.

## Author Contributions

L-DC and Z-WH performed most of the experiments with assistance and wrote the manuscript. Y-ZH, J-FH, and Z-PZ contributed to the data analyses. X-JL edited the manuscript. All authors have read and agreed to the published version of the manuscript.

## Conflict of Interest

The authors declare that the research was conducted in the absence of any commercial or financial relationships that could be construed as a potential conflict of interest.

## Supplementary Material

Supplementary Figure 1 Quality control of UHPLC-Q-TOF/MS analysis. **(A)** Total ion chromatograms in positive ion mode and **(B)** negative ion mode for quality control samples. The retention time and response strength of each chromatographic peak almost overlap. **(C)** Pearson correlation analysis of quality control samples in positive ion mode and **(D)** negative ion mode. Correlation coefficients between quality control samples are all above 0.9. **(E)** RSD of quality samples in positive ion mode and **(F)** negative ion mode. More than 80% of peaks have an RSD < 0.3. **(G)** Hotelling’s T2 analyses of all samples in positive ion mode and **(H)** negative ion mode. All the samples are within the 99% confidence interval. **(I)** Multivariate control chart analysis of quality control samples in positive ion mode and **(J)** negative ion mode. The score values of all samples fall within ± 3SD.

## Abbreviations

ALT: alanine aminotransferase
AST: aspartate aminotransferase
ESI: electrospray ionization
CIH: chronic intermittent hypoxia
HE: hematoxylin–eosin
IH: intermittent hypoxia
KEGG: Kyoto Encyclopedia of Genes and Genomes
NAFLD: non-alcoholic fatty liver disease
NC: normal control
OSA: obstructive sleep apnea
OPLS-DA: orthogonal partial least-squares discriminant analysis
PCA: principal component analysis
SD: standard deviation
UHPLC/Q-TOF MS: ultra-high performance liquid chromatography/quadrupole time-of-flight mass spectrometry
VIP: variable importance for the projection.
