# Supplementary figures and images for: Untargeted Metabolomic Profiling of Liver in a Chronic Intermittent Hypoxia Mouse Model
Source: Front Physiol. 2021 Jul 8;12:701035. doi: 10.3389/fphys.2021.701035 (PMC8298499; doi:10.3389/fphys.2021.701035)

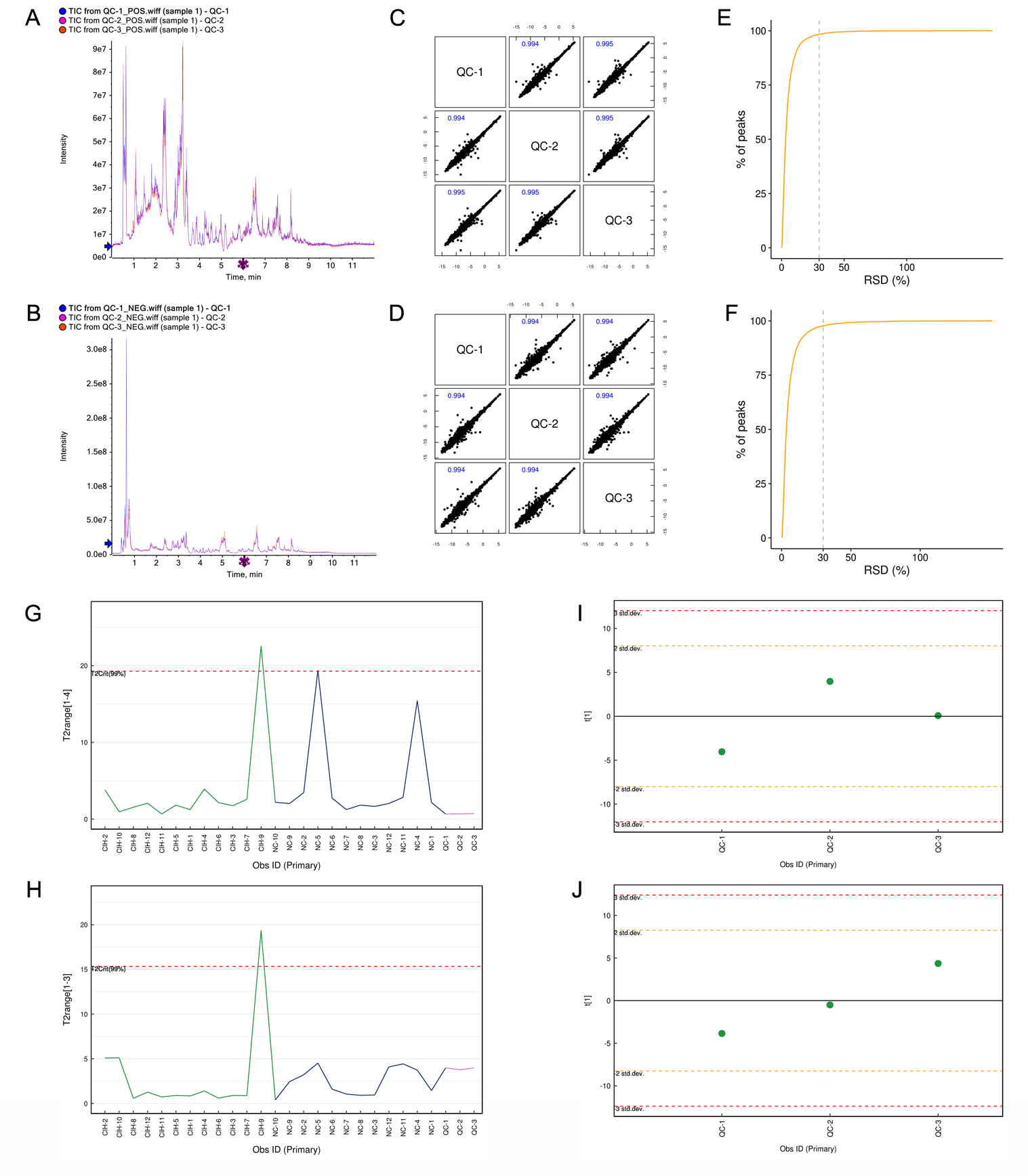

Supplement: Supplementary Figure 1 — Quality control of UHPLC-Q-TOF/MS analysis. (A) Total ion chromatograms in positive ion mode and (B) negative ion mode for quality control samples. The retention time and response strength of each chromatographic peak almost overlap. (C) Pearson correlation analysis of quality control samples in positive ion mode and (D) negative ion mode. Correlation coefficients between quality control samples are all above 0.9. (E) RSD of quality samples in positive ion mode and (F) negative ion mode. More than 80% of peaks have an RSD < 0.3. (G) Hotelling’s T2 analyses of all samples in positive ion mode and (H) negative ion mode. All the samples are within the 99% confidence interval. (I) Multivariate control chart analysis of quality control samples in positive ion mode and (J) negative ion mode. The score values of all samples fall within ± 3SD. [file Image_1.TIF]
